# Protection of the Queuosine Biosynthesis Enzyme QueF from Irreversible Oxidation by a Conserved Intramolecular Disulfide

**DOI:** 10.3390/biom7010030

**Published:** 2017-03-16

**Authors:** Adeba Mohammad, Adriana Bon Ramos, Bobby W. K. Lee, Spencer W. Cohen, Maryam K. Kiani, Dirk Iwata-Reuyl, Boguslaw Stec, Manal A. Swairjo

**Affiliations:** 1 Graduate College of Biomedical Sciences, Western University of Health Sciences, 309 E. Second Street, Pomona, CA 91766, USA; amohammad@westernu.edu (A.M.); keemia@gmail.com (M.K.K.); 2 Department of Chemistry, Portland State University, P.O. Box 751, Portland, OR 97207, USA; bonramos@pdx.edu (A.B.R.); bowklee@gmail.com (B.W.K.L.); cohens@oregonstate.edu (S.W.C.); iwatard@pdx.edu (D.I.-R.); 3 Department of Chemistry and Biochemistry, San Diego State University 5500 Campanile Drive, San Diego, CA 92182, USA; bog.stec.2010@gmail.com

**Keywords:** tRNA modification, oxidoreductase, tunneling fold

## Abstract

QueF enzymes catalyze the nicotinamide adenine dinucleotide phosphate (NADPH)-dependent reduction of the nitrile group of 7-cyano-7-deazaguanine (preQ_0_) to 7-aminomethyl-7-deazaguanine (preQ_1_) in the biosynthetic pathway to the tRNA modified nucleoside queuosine. The QueF-catalyzed reaction includes formation of a covalent thioimide intermediate with a conserved active site cysteine that is prone to oxidation in vivo. Here, we report the crystal structure of a mutant of *Bacillus subtilis* QueF, which reveals an unanticipated intramolecular disulfide formed between the catalytic Cys55 and a conserved Cys99 located near the active site. This structure is more symmetric than the substrate-bound structure and exhibits major rearrangement of the loops responsible for substrate binding. Mutation of Cys99 to Ala/Ser does not compromise enzyme activity, indicating that the disulfide does not play a catalytic role. Peroxide-induced inactivation of the wild-type enzyme is reversible with thioredoxin, while such inactivation of the Cys99Ala/Ser mutants is irreversible, consistent with protection of Cys55 from irreversible oxidation by disulfide formation with Cys99. Conservation of the cysteine pair, and the reported in vivo interaction of QueF with the thioredoxin-like hydroperoxide reductase AhpC in *Escherichia coli* suggest that regulation by the thioredoxin disulfide-thiol exchange system may constitute a general mechanism for protection of QueF from oxidative stress in vivo.

## 1. Introduction

**Figure 1:**
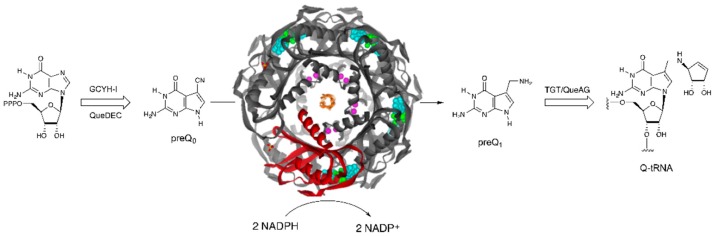
The QueF-catalyzed reaction in the queuosine biosynthesis pathway.

QueF is the nicotinamide adenine dinucleotide phosphate (NADPH)-dependent nitrile reductase that functions in the biosynthetic pathway of the tRNA-modified nucleoside queuosine (Q, [1]), a 7-deazaguanosine nucleoside found at the wobble position of bacterial and eukaryotic tRNAs possessing the GUN anticodon (those encoding for Tyr, His, Asp and Asn) [2]. QueF catalyzes the NADPH-dependent 4-electron reduction of the nitrile group of the pathway intermediate 7-cyano-7-deazaguanine (preQ_0_) to 7-aminomethyl-7-deazaguanine (preQ_1_), the last intermediate in the tRNA-independent portion of the pathway [1] (Figure 1). PreQ_1_ is subsequently inserted into the tRNA by the enzyme tRNA-guanine transglycosylase [3], and the remainder of the pathway occurs on the tRNA [4,5]. Although Q is essential for translational fidelity and efficiency in both Bacteria and Eukarya [6,7,8,9], de novo Q biosynthesis, hence the entire tRNA-independent portion of the Q pathway, including the QueF step, occurs only in Bacteria [4,10]. The QueF catalyzed reaction is the only example of biological nitrile reduction known to date.

**Figure 2:**
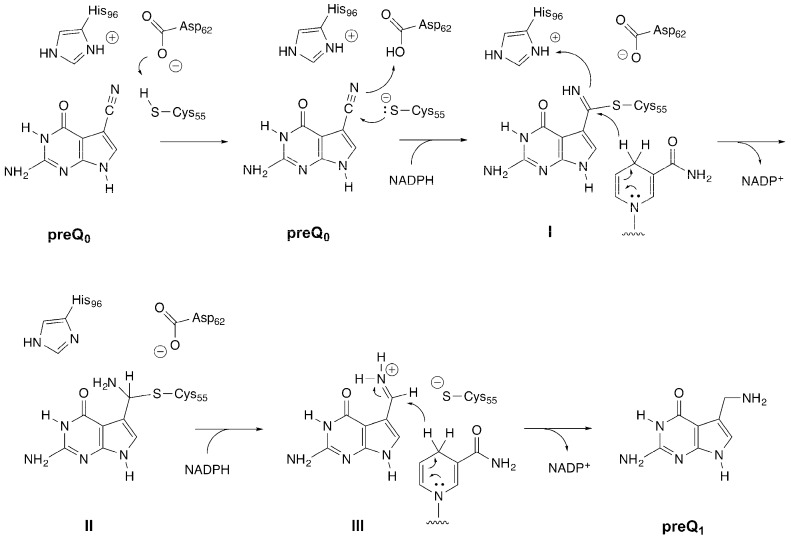
The proposed catalytic mechanism of the QueF catalyzed reaction (amino acid numbering based on the *Bacillus subtilis* enzyme).

QueF enzymes from *Bacillus subtilis* [1,11], *Vibrio cholera* [12], *Geobacillus kaustophilus* [13], and *Escherichia coli* [14,15] have been biochemically characterized, and a number of mechanistic investigations have been reported [11,16,17,18]. The reaction begins with the binding of preQ_0_ and its reaction with the thiol of an active site cysteine (Cys55 in the *B. subtilis* enzyme) [11,16] to form a covalent thioimide intermediate (Figure 2, I). This intermediate is very stable, with a rate constant for decomposition of ~5 × 10^−6^ s^−1^ [16]. NADPH then binds and delivers the first hydride equivalent to generate a covalent thiohemiaminal (Figure 2, II) intermediate. After dissociation of NADP^+^ and binding of the second equivalent of NADPH, II breaks down to form an imine (Figure 2, III), which is then reduced to preQ_1_.

**Figure 3:**
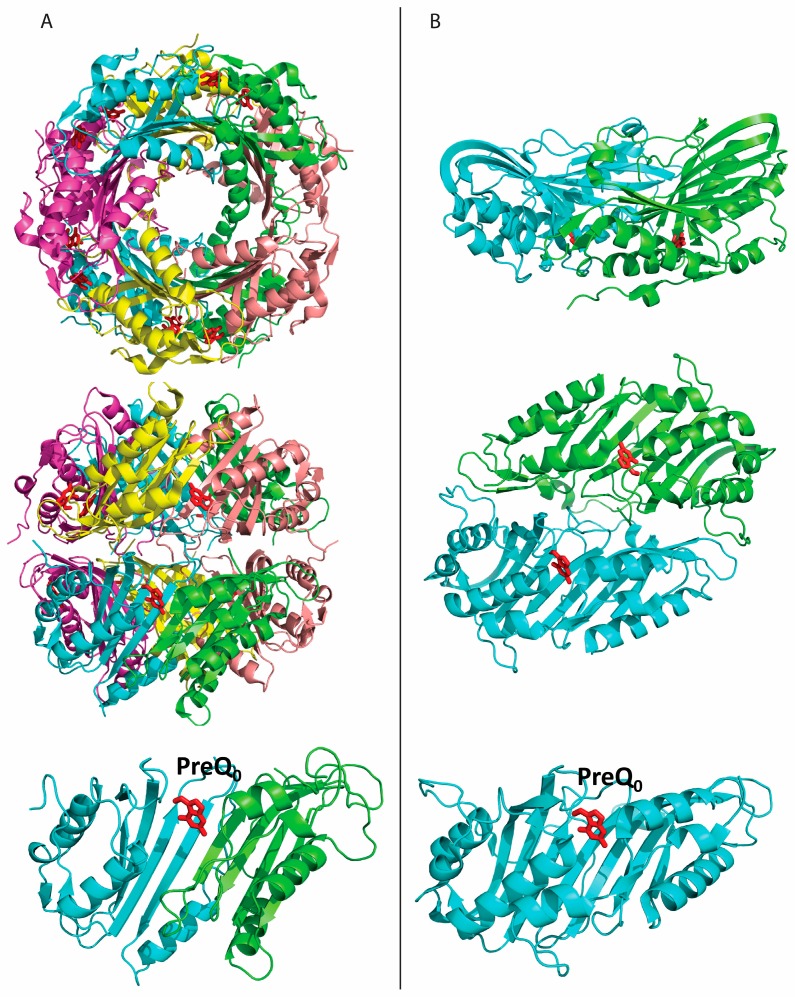
Structural overview of the two QueF subfamilies. Crystal structures of the *B. subtilis* QueF homodecamer (Protein Data Bank (PDB) ID 4F8B, [16]) as representative of the unimodular QueF subfamily (**A**) and of the *Vibrio Cholerae* QueF homodimer (PDB ID 3S19) as representative of the bimodular subfamily (**B**). Top and middle: view of the biological multimer down the central tunnel of *B. subtilis* QueF and the analogous view in *V. cholerae* QueF. Bottom: view of the active site at the interface between two T-fold subunits in *B. subtilis* QueF and at the inter-domain interface within a single subunit in *V. cholerae* QueF. Subunits are shown as ribbon diagrams in different colors. Bound preQ_0_ molecules are shown in a red stick model.

QueF belongs to the tunnelling fold (T-fold) structural superfamily of purine/pterin binding proteins. T-fold proteins share a small domain (T-fold domain) that consists of a four-stranded antiparallel β sheet and two α helices between the 2nd and 3rd strands (ββαββ) laying on one face of the β sheet [19]. QueF enzymes fall into two subfamilies: the unimodular subfamily, exemplified by *B. subtilis* QueF, is comprised of proteins that are constructed from subunits possessing a single T-fold domain, whereas the bimodular subfamily—e.g., *V. cholerae* and *E. coli* QueF—is comprised of proteins that are constructed from subunits possessing two weakly homologous tandem T-fold domains [1]. *B. subtilis* QueF, a ~160 amino acid protein with a single T-fold domain, is a homodecamer of two head-to-head facing pentamers, each composed of a cyclic arrangement of monomeric T-fold subunits, forming a tunnel in the center (Figure 3A) [16]. The homodecamer contains 10 active sites at the intersubunit interfaces. *V. cholera* QueF, a ~280 amino acid polypeptide with two tandem T-fold domains, exists as a homodimer with two active sites located at the interfaces between the two T-fold domains within the monomeric subunits (Figure 3B) [12]. This quaternary structure does not exhibit a central tunnel, hence *V. cholerae* QueF does not conform to the canonical T-fold assembly. Both QueF subfamilies harbor a conserved QueF motif embedded in a helix flanking the active site [1,16]. The QueF motif E(S/L)K(S/A)hK(L/Y)(Y/F/W (where h is a hydrophobic residue) is characteristic of QueF enzymes and contains residues responsible for NADPH binding. The active sites in both subfamilies contain an invariant glutamate residue (Glu97 and Glu234 in *B. subtilis* QueF and *V. cholerae* QueF, respectively) that anchors the preQ_0_ substrate in the active site, and an invariant cysteine residue (Cys55 and Cys194 in *B. subtilis* QueF and *V. cholerae* QueF, respectively) that forms a thioimide intermediate with preQ_0_ [11,12,16]. In unimodular QueF, these conserved elements are contained within the same T-fold domain [16], whereas in bimodular QueF, the QueF motif and the active site Cys and Glu are separated in the two domains and join together in the tertiary structure to form an interdomain active site [12].

The catalytic cysteine of QueF has been shown to be prone to oxidation in vivo in the proteomes of several *Bacillus* species when exposed to oxidative stress from sodium hypochlorite [20]. It has also been shown that QueF interacts in vivo with the thioredoxin-like alkyl hydroperoxide reductase AhpC in *E. coli*, suggesting that the enzyme may be regulated by the thioredoxin disulfide-thiol exchange system [21]. Here, we present crystallographic and biochemical evidence that *B. subtilis* QueF is protected from irreversible oxidation by a conserved intramolecular disulfide between the catalytic Cys55 and a second cysteine (Cys99) located in a helix lining the active site, and that oxidative inactivation of the enzyme is reversible with thioredoxin. Bioinformatic and phylogenetic analyses of the two QueF subfamilies reveal a conservation pattern of the disulfide that is consistent with a biological role in adaptation to oxidative stress environments.

## 2. Results and Discussion

### 2.1. Activity of B. subtilis QueF Mutants

Both of the Cys99 mutants exhibited robust activity (Table 1), while the Glu97Gln mutant displayed low, but measurable, activity (~2% of wild-type). Given the significantly decreased activity of this mutant, we carried out a full steady-state analysis to determine the kinetic constants, which revealed a *K*_M_(preQ_0_) = 67 μM and *k*_cat_ = 0.036/min. For comparison, the wild-type enzyme exhibits a *K*_M_(preQ_0_) = 0.237 μM and *k*_cat_ = 0.66/min [11]. The much larger impact on *K*_M_ (280-fold) as compared to *k*_cat_ (18-fold) is consistent with the putative role of Glu97 in substrate binding [1,16].

### 2.2. Formation of an Active-Site Disulfide in a Substrate-Free Mutant of B. subtilis QueF

**Figure 4:**
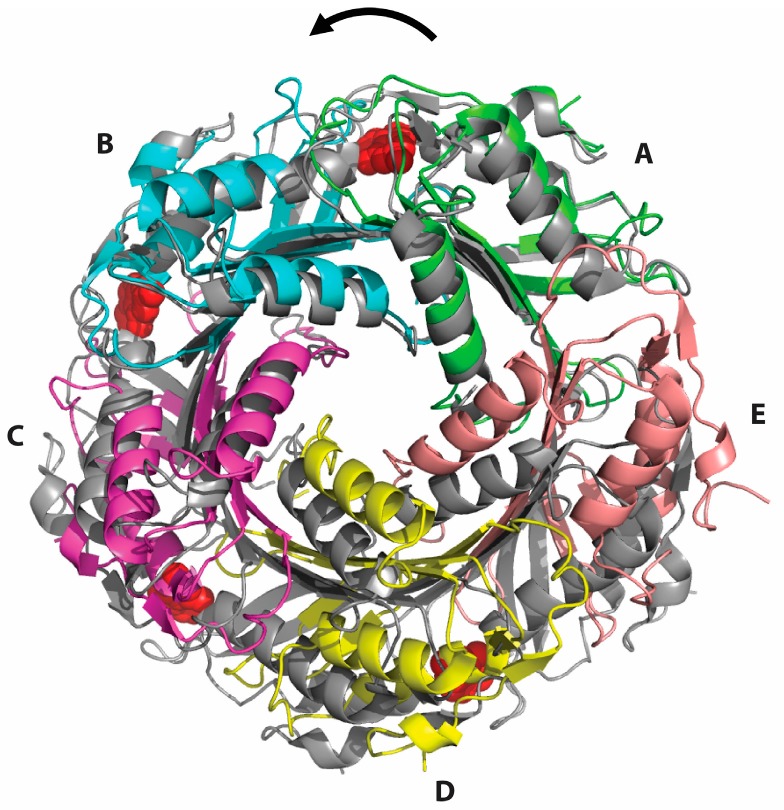
Superposition of the crystal structures of the substrate-free Glu97Gln mutant of *B. subtilis* QueF (colors) with the substrate-bound wild-type enzyme as a thioimide intermediate (grey) generated by optimizing alignment of subunits **A** from both structures. Successive shifts in the positions of subunits **B**–**E** result in tightening of the decamer in a counterclockwise screw fashion indicated by the arrow. For clarity, only one pentamer is shown. Bound preQ0 molecules in the wild-type structure are shown in red.

**Figure 5:**
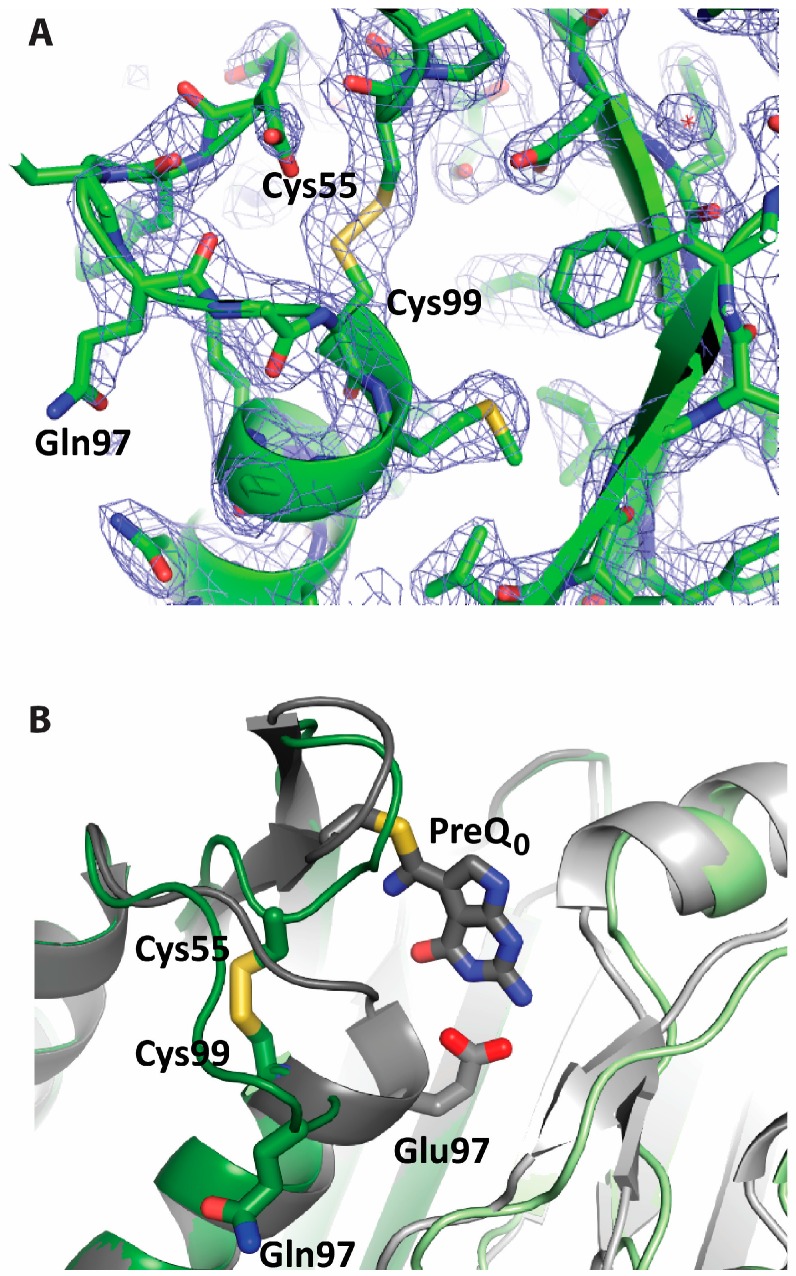
View of the intramolecular disulfide bridge in the active site of the Glu97Gln mutant of *B. subtilis* QueF. (**A**) 2*Fo*-*Fc* electron density map (2.5 Å, contour 1.2 σ), superposed on the refined model, in the active site region; (**B**) superposition in the active site region of the crystal structures of the Glu97Gln mutant (green) and the wild-type enzyme thioimide intermediate (grey, PDB ID 4F8B) showing conformational changes associated with disulfide formation. The two interface subunits are shown in two shades of color. Bound preQ_0_ in the active site of wild-type QueF and key active site residues are shown in stick diagram and labeled.

The Glu97Gln mutant enzyme crystallized in the same space group (P3_2_21) as previously determined for the wild-type enzyme and the Cys55Ala mutant, both bound to preQ_0_ [16]. The refined structure contains five subunits (A–E) in the crystallographic asymmetric unit and exhibits good geometry and an R factor below 0.2 (Table 2). The overall structure shows a homodecamer formed by two pentameric subunits organized in a face-to-face manner through coordination of the C-terminal tails via divalent metal ions, similar to the preQ_0_-bound structures. However, the Glu97Gln mutant structure is substantially more symmetric, and represents the substrate- and cofactor-free form of the enzyme (here referred to as the apo form) as no difference electron density corresponding to the substrate, cofactor or the product is seen in any of the ten active sites in the homodecamer. Superposition of the Glu97Gln mutant structure with any of the preQ_0_-bound structures (wild-type or Cys55Ala) reveals significant tightening of the homodecamer in the apo form relative to the preQ_0_-bound form (Figure 4). Successive counterclockwise shifts in the positions of the subunits manifests as a net rotation of subunit E by 25° around the tunnel five-fold axis and translation of its center of mass along the axis by 17 Å relative to its position in the preQ_0_-bound structures. The most striking difference is the formation of a disulfide bridge in all the active sites between the catalytic Cys55 and Cys99 located in the N-terminal turn of the second helix of the tunnel fold, the helix lining the inter-subunit interface (Figure 5). Disulfide formation is accompanied by full unwinding of the N-terminal turn of the helix, indicating conformational flexibility of the active sites. These disulfides are apparently very stable, given that reducing agents were included in the purification and crystallization buffers.

The observed disulfides do not seem to serve a structural role since they do not occur in the substrate-bound QueF structures. To confirm this interpretation, we generated the Cys99Ala and Cys99Ser mutants of the enzyme and tested the effect of the mutation on enzyme activity. Both mutants were fully active (Table 1), indicating that the proteins were structurally intact and that Cys99 does not play a catalytic role.

Furthermore, the structural changes seen in the active sites in association with disulfide formation suggest that the disulfides may serve a regulatory function as allosteric disulfides [22]. Disulfide bonds in proteins have been functionally classified based on the geometry and dihedral strain of the bond as defined by the sign and magnitude, respectively, of the five dihedral angles, χ_1_, χ_2_, χ_3_, χ_2_′, χ_1_′, which make up the bond [22,23]. In the present structure, the χ_1_ and χ_1_′ dihedral angles are defined by the atom groups N-C_α_-C_β_-S_γ_ and N′-C_α_′-C_β_′-S_γ_′, respectively, where unprimed and primed atoms belong to the Cys55 and Cys99 halves of the disulfide bond, respectively. χ_2_ and χ_2_′ are the dihedral angles defined by C_α_-C_β_-S_γ_-S_γ_′ and C_α_′-C_β_′-S_γ_′-S_γ_, respectively, and χ_3_ is defined by C_β_-S_γ_-S_γ_′-C_β_′. We analyzed the disulfide bond geometries in the QueF Glu97Gln mutant structure using the Disulfide Bond Dihedral Angle Energy Server (http://services.mbi.ucla.edu/disulfide/). All of the disulfides in the protein decamer exhibit dihedral angles with the signs −,−,−,+,− for χ_1_, χ_2_, χ_3_, χ_2_′, χ_1_′, respectively (Table 3), indicating a minus left-handed hook (–LHHook) geometry characteristic of regulatory disulfides [24]. These angles and the calculated disulfide strain energy of ~13–19 kJ/mol suggest an allosteric regulatory function, rather than a catalytic or structural function, for the active site disulfides of QueF [22].

### 2.3. Conservation of Disulfide-Forming Cysteines in QueF Proteins

**Figure 6:**
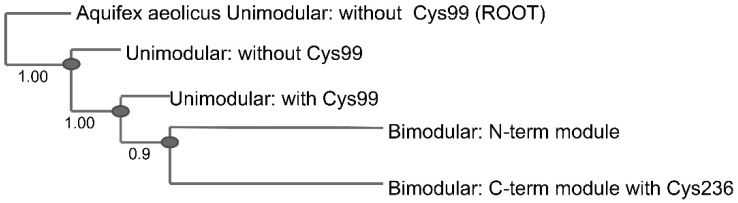
Phylogenetic tree of QueF proteins. The tree was rooted to *Aquifex aeolicus* QueF sequence (unimodular QueF with no Cys99). Numbers indicate the posterior probability value.

Multisequence alignment of 2074 unimodular and 1375 bimodular QueF non-redundant sequences from the National Center for Biotechnology Information (NCBI) database revealed that Cys99 (*B. subtilis* residue numbers) is conserved in ~61% of the unimodular sequences (Table 4). An additional 22% of the sequences lack Cys99 but harbor an alternative cysteine at position 53 (in *B. subtilis* residue numbers), located in the active site loop in spatial proximity to Cys55 (based on 3D homology models, Supplementary Figure S1), bringing the total conservation of a potential disulfide forming backdoor cysteine to ~83%. In contrast, in bimodular QueF, the cysteine residue in a homologous position to Cys99 is Cys236 (in *V. cholerae* residue numbers), and is conserved in 100% of the bimodular sequences. Utilizing all sequences, a phylogenetic tree was generated using a multisequence alignment of unimodular QueF with the separated N- and C-terminal modules of bimodular QueF (Figure 6). The tree was rooted to the QueF sequence from the ancient bacteria *Aquifex aeolicus*. The phylogenetic distribution indicates that unimodular QueF lacking Cys99 is the oldest variant. Gain of Cys99 occurred later in evolution but before the gene duplication event leading to bimodular QueF. Through divergent evolution, the catalytic cysteine and Cys236 were retained in the C-terminal module of bimodular QueF, and were lost from the N-terminal module.

### 2.4. Disulfide-Mediated Protection of QueF from Irreversible Oxidation In Vitro

Regulatory/allosteric active site disulfides in enzymes are posttranslational thiol modifications that regulate enzyme function in a nonenzymatic way by triggering changes in the intra- or intermolecular structure of the protein in response to a signal such as oxidative stress [22]. A common cellular mechanism for disulfide-mediated redox sensing and response is the thioredoxin disulfide-thiol exchange system [25]. QueF has been found to interact with the thioredoxin-like alkyl hydroperoxide reductase protein C (AhpC) in vivo [21], prompting us to hypothesize that QueF may be a substrate of thioredoxin and that the disulfide protects the enzyme from irreversible oxidation. To test this hypothesis, we investigated the potential of thioredoxin to restore the activities of wild-type *B. subtilis* QueF and of the mutants Cys99Ala/Ser after inactivation with hydrogen peroxide.

**Figure 7:**
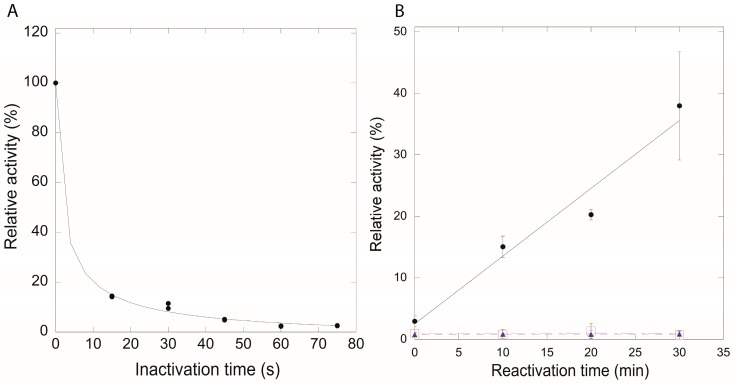
(**A**) A sample time course for oxidative inactivation of wild-type QueF; and (**B**) time course for thioredoxin dependent reactivation of wild-type QueF (filled circles), Cys99Ala (filled triangles), and Cys99Ser (open squares) mutants of QueF.

Thus, in vitro peroxide assays were conducted using the wild-type *B. subtilis* QueF and mutants Cys99Ala/Ser. Both wild-type QueF and the Cys99Ala/Ser mutants were inactivated rapidly with H_2_O_2_ (Figure 7A) presumably due to oxidation of the catalytic Cys55. However, the H_2_O_2_ induced inactivation of the wild-type enzyme was reversible with thioredoxin (Figure 7B), while inactivation of the Cys99Ala/Ser mutants was irreversible, consistent with the hypothesis that Cys55 is protected from irreversible oxidation by disulfide formation with Cys99 in the wild-type enzyme.

It has been demonstrated that the catalytic Cys55 of QueF is prone to oxidation in vivo in bacilli exposed to sodium hypochlorite [20] and can be protected from overoxidation by S-bacillithiolation, a posttranslational thiol modification constituting a biological redox control mechanism in which protein thiols form mixed disulfides with bacillithiol, an α-anomeric glycoside of l-cysteinyl-d-glucosamine with L-malic acid and a major low-molecular-weight thiol redox buffer in Bacillus species and other Gram-positive bacteria. Our results suggest that thioredoxin-responsive active site disulfides may constitute a second and more widely spread biological mechanism for protection of QueF from irreversible oxidation in vivo. Consistent with this proposal, we observe a higher conservation of the disulfide forming cysteine in QueF proteins from pathogenic bacteria where oxidative stress imposed by the host immune response is high. For example, upon inspecting QueF sequences from 164 human and plant bacterial pathogens, only seven sequences lack Cys99/Cys236, and six of them harbor the potential alternative Cys53 (Supplementary Table S1). Conversely, when considering a sample of 333 QueF sequences that lack Cys99 and any alternative backdoor cysteine, all but five are from non-pathogens. Lastly, the strict conservation of Cys236 in bimodular QueF is consistent with the prevalence of the bimodular QueF subfamily in pathogenic bacteria (Supplementary Table S1).

## 3. Materials and Methods

### 3.1. Mutagenesis of QueF

Mutagenesis was carried out with the QuikChange XL (Stratagene, La Jolla, CA, USA) or QuikChange II (Agilent, Santa Clara, CA, USA) kits. The sequences of the primers used for the construction of the mutant plasmids were as follows:

E97Q(sense)
5’-GTTCATGATGATATTCATGCAGTCCTTGTGGAAGTCAC-3’
E97Q(antisense)
5’-GGTGACTTCCACCAGGACTGCATGAATATCATCATGAACG-3’
C99S(sense)
5’-GGTGACTTCCACGAGGACAGCATGAATATCATCATGAACG-3’
C99S(antisense)
5’- CGTTCATGATGATATTCATGCTGTCCTCGTGGAAGTCACC-3’
C99A(sense)
5’-GGTGACTTCCACGAGGACGCCATGAATATCATCATGAACG-3’
C99A(antisense)
5’- CGTTCATGATGATATTCATGGCGTCCTCGTGGAAGTCACC-3’.

The *queF* gene in the pET-30Xa vector [1] was used as a template to generate the single mutants. The PCR protocol consisted of an initial hold at 94 °C for 45 s, followed by 18 cycles of 94 °C for 45 s, 55 °C for 60 s, and 68 °C for 8 min. After 18 cycles, the reaction mixtures were kept at 4 °C. *Dpn1* (1 µL, 10 U/µL, Fermentas) was added and the reaction mixture was incubated at 37 °C for 1 h before the plasmid was transformed into ultracompetent *E. coli* (DH5α) cells. Single colonies grown overnight on kanamycin containing (30 µg·mL^−1^) agar plates were selected and cultured in 3 mL Luria-Bertani media containing 30 µg·mL^−1^ for 7 h. The plasmid DNA was purified using the Qiagen miniprep kit (Qiagen, Germantown, MD, USA), and the mutated genes sequenced to verify the mutation and the otherwise unaltered DNA sequence. For protein expression, the mutant plasmids were transformed into the *E. coli* BL21(DE3) cell line. The expression and purification of the QueF mutant proteins were carried out as previously described for the wild-type protein [11,26].

### 3.2. Activity Assays of Glu97Gln and Cys99Ala/Ser Mutants

Standard assays for measuring the enzymatic activity of wild-type and mutant QueF enzymes followed the oxidation of NADPH by ultraviolet-visible spectrophotometry (UV-Vis) as described previously [11]. For determining the kinetic parameters of the Glu97Gln mutant, the fluorescence assay based on the decomposition of product NADP^+^ was employed as previously described [11].

### 3.3. Crystallization, X-ray Data Collection and Crystal Structure Determination

The Glu97Gln QueF mutant was crystallized in the absence of preQ_0_ using the vapor diffusion method at 293.15 K. Briefly, a sample containing 4 mg/mL protein, in 50 mM Tris pH 7.5, 50 mM KCl, 1 mM MgCl_2_ and 1 mM β-mercaptoethanol (BME) was prepared. Hanging drops were setup by mixing equal volumes of sample and reservoir solution containing 19% polyethylene glycol monomethyl ether 550, 43 mM imidazole-Cl (pH 6.8), 53 mM imidazole (pH 8.2), 30 mM CaCl_2_, and 4% dimethyl sulfoxide. Rhomb shaped crystals appeared in two days and were harvested and flash frozen in liquid nitrogen without use of additional cryoprotectant. The X-ray data were collected using synchrotron radiation at the Stanford Synchrotron Radiation Lightsource, beamlines 7-1. The data were processed using HKL2000 [27]. The structure was determined using the molecular replacement Bayesian protocol in the Phaser crystallographic software (version 2.7.17) [28] and using the structure of a single *B. subtilis* QueF monomer (from PDB ID 4FGC) as a search model. The presence of the disulfide in the structure was confirmed by difference Fourier methods using model phases with Cys55 and Cys99 deleted from the model (omit map). The structure was refined using Refmac (version 5.8.0135) [29] and Coot (version 0.8.2) [30]. The X-ray data and 3D coordinates have been deposited in the PDB under accession ID 5UDG.

### 3.4. Sequence Analysis

A BLASTp search of the NCBI protein database, conducted using the sequence of *B. subtilis* QueF, yielded a non-redundant set of ~3500 sequences. The sequences were aligned using the Multiple Alignment Using Fast Fourier Transform (MAFFT) program within the Jalview software [31]. Sequences were checked for the simultaneous presence of the active site residues defining the QueF family. These features include Cys55/194 (*B. subtilis* residue numbers/*V. cholerae* residue numbers), Asp62/201, Glu97/234, and the QueF motif including Glu78/94 [1,16]. Sequences lacking any of these features were considered non-QueF and were excluded from the analysis. The sequences were divided into the two subfamilies, unimodular (<200 residues with a single T-fold domain) and bimodular (>200 residues with tandem T-folds) and further examined for the presence of Cys99/236. For sequences lacking Cys99/236, 3D homology models were produced using Phyre2 (version 2.0) [32] and inspected for the presence of alternative cysteine residues in the active site region. Phylogenetic analysis was conducted in the program TOPALi (version 2.5) [33] and the phylogenetic tree was calculated using MrBayes (version 3.2) [34].

### 3.5. H_2_O_2_ Oxidation of Wild-Type QueF and Cys99Ala/Ser Mutants

A 120 μL stock QueF solution containing 100 mM phosphate (pH 6.5), 50 mM KCl, 20 mM MgCl_2_, and 36 μM protein was prepared, and a 20 μL aliquot was removed. To the remaining stock solution was added 5 μL of a solution of 1 mM hydrogen peroxide and this was allowed to react. At time-points throughout the reaction (15, 30, 45, 60 or 75 s), a 20 μL aliquot was removed from the reaction and added to a solution (80 μL) containing 12 units of catalase, and 1 mM dithiothreitoland mixed thoroughly to quickly quench the unreacted hydrogen peroxide. The quenched solution was then transferred to a microcuvette and preQ_0_ and NADPH were added to a final concentration of 100 μM and 180 μM, respectively, and the absorbance of the reaction at 340 nm was monitored over a period of 20 min.

### 3.6. Activity Recovery of Oxidized QueF Enzymes

Wild type and Cys99Ala/Ser mutant enzymes of QueF were oxidized with H_2_O_2_ for 45 s under the conditions described above. The oxidation reactions were then quenched with a solution containing 12 units of catalase and 5 equivalents of thioredoxin. At time-points throughout the reaction (10, 20, and 30 min), a 20 μL aliquot of the solution was transferred to a microcuvette containing 100 μM preQ_0_ and 180 μM NADPH. The absorbance at 340 nm was monitored over a period of 20 min to determine the initial velocity. To ensure accurate measurement of recovered activity, a control experiment was performed in which the activity of each enzyme was measured after oxidation as described above to ensure that each had been rendered inactive prior to treatment with thioredoxin.

## 4. Conclusions

QueF is protected from irreversible oxidation by a conserved intramolecular disulfide that can be reduced by thioredoxin, and regulation by the thioredoxin disulfide-thiol exchange system may constitute a general mechanism for protection of QueF from oxidative stress in vivo.

## Figures and Tables

**Table 1:** Relative catalytic activity of wild-type and mutant QueF enzymes.

Enzyme	Relative Activity ^1^ (%)
Wild-type QueF	100 ± 4
Cys99Ala	80 ± 4
Cys99Ser	74 ± 3
Glu97Gln	1.9 ± 0.1

^1^ Relative activity reflects the average initial velocities for each enzyme (determined via ultraviolet-visible spectrophotometry time course assays) divided by the average initial velocity for the wild-type enzyme. Standard errors for the initial velocity measurements of specific enzymes ranged from 3% to 4.5%.

**Table 2:** X-ray data collection parameters and structure refinement statistics.

Data Collection:	Value
Space group	P3_2_21
Unit cell (Å)	87.31, 87.31, 196.73
Wavelength (Å)	1.12709
Resolution range (Å)	50–2.5 (2.54–2.50) ^1^
Completeness (%)	98.0 (92.5)
Redundancy	5.0 (3.0)
R_merge_, R_pim_ (%) ^2^	0.087, 0.060 (0.630, 0.627)
<I/σ(I)>	13.10 (1.17)
**Refinement:**	
Number of reflections	
Working/free	28,753/1466 (1937/109)
Number of atoms	
Total	6417
Water/Mg^2+^	285/7
PEG	37
R-cryst ^3^/R-free ^4^ (%)	0.189/0.257 (0.303/0.409)
Rmsd bond lengths (Å)	0.019
Rmsd bond angles (°)	2.007
Wilson B-factor (Å2)	50.2
Average B-factor	
Protein	45.5
Metals	79.7
Water	46.76
Ramachandran Plot (%)	
Favored	94.0
Allowed	4.5 ^5^

^1^ Highest-resolution shell information in parentheses; ^2^ R_merge_ = Σ|I_obs_ − <I>|/ΣI_obs_, R_pim_ = (Σ_h_ [1/(n_h_ − 1)]^1/2^ Σ_i_ | <I(h)> − I(h)_i_ |)/Σ_h_ Σ_i_ I(h)_I_; ^3^ Crystallographic R-factor = Σ||F_obs_| − |F_calc_||/Σ|F_obs_|; ^4^ The free R-factor was monitored with 5% of the data excluded from refinement; ^5^ The ten outlier residues are three glycine residues, five metal binding aspartate residues, and two N-terminal residues. PEG: polyethylene glycol.

**Table 3:** Dihedral angles, bond lengths, and strain energies of the Cys55-Cys99 disulfides in the crystal structure of *B. subtilis* Glu97Gln QueF mutant.

Subunit	χ_1_ (°)	χ_2_ (°)	χ_3_ (°)	Bond Length (Å)	χ_2_′ (°)	χ_1_′ (°)	Disulfide Strain Energy (kJ/mol)
A	−59.85	−126.76	−105.12	2.03	176.01	−66.90	15.023
B	−56.90	−123.68	−89.05	2.03	171.20	−81.83	16.120
C	−59.70	−116.71	−102.91	2.05	168.10	−63.57	14.772
D	−60.93	−127.32	−79.75	2.07	173.65	−89.46	18.584
E	−55.16	−120.73	−94.72	2.04	172.66	−72.34	13.938

**Table 4:** Conservation of Cys99 in unimodular QueF and the homologous residue Cys236 in bimodular QueF. NA: Not applicable.

	Unimodular QueF	Bimodular QueF
Total sequences	2074	1375
% with disulfide forming cysteine (Cys99 in unimodular QueF, Cys236 in bimodular QueF)	61%	100%
% with any potentially disulfide forming cysteine (Cys99 or Cys53 in unimodular QueF)	83%	NA

## Supplementary Materials

The following are available online at http://www.mdpi.com/2218-273X/7/1/30/s1. Figure S1: 3D homology model of unimodular QueF, which lacks the disulfide forming Cys99, but contains Cys53 in proximity to the catalytic Cys55 in the active site loop. The model was generated using the *Aquifex aeolicus* QueF sequence and the Phyer2 server (http://www.sbg.bio.ic.ac.uk/phyre2/html/page.cgi?id=index). Table S1: Classification of 164 bacterial pathogens based on the subfamily that their QueF proteins belong to and the presence/absence of the disulfide forming Cys99 (and the homologous Cys236 in bimodular QueF) in the protein sequence.
