# Supplementary material for: Protection of the Queuosine Biosynthesis Enzyme QueF from Irreversible Oxidation by a Conserved Intramolecular Disulfide
Source: Biomolecules. 2017 Mar 16;7(1):30. doi: 10.3390/biom7010030 (PMC5372742; doi:10.3390/biom7010030)
Supplement: Supplementary file 1 [file biomolecules-07-00030-s001.pdf]

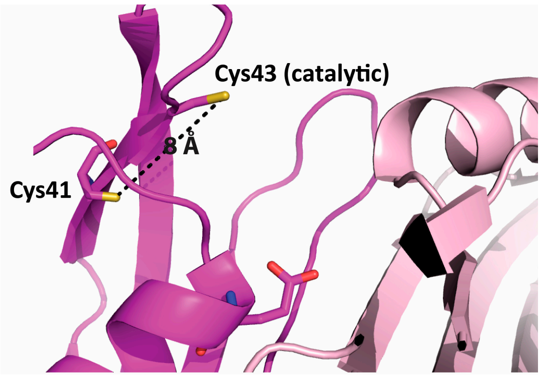


**Figure S1.** Active site region in a 3D homology model of a unimodular QueF protein that lacks a cysteine homologous to the disulfide Cys99 of *Bacillus subtilis* QueF, but contains an alternative backdoor cysteine located in proximity to the catalytic cysteine in the active site loop. The model is of the *Aquifex aeolicus* QueF protein and shows the catalytic cysteine and the putative alternative backdoor cysteine in *A. aeolicus* residue numbers. The two interface subunits making the active site are shown in two shades of magenta.

**Table S1.** Examples of bacterial pathogens classified based on the subfamily their QueF proteins belong to and the presence/absence of the disulfide forming Cys99 (and the homologous Cys236 in bimodular QueF) in the protein sequence.

| **Human Pathogens** | | | | **Plant Pathogens** | | | |
|---|---|---|---|---|---|---|---|
| **Uni No Cys99** | **Uni No Cys99 and No Cys53** | **Uni Cys99** | **Bi Cys236** | **Uni No Cys99** | **Uni Cys99** | **Bi Cys236** | **Total Sequences** |
| **5** | **1** | **23** | **56** | **2** | **13** | **41** | **140** |
| *Campylobacter jejuni* | *Leptospira interrogans* | *Veillonella* | *Acinetobacter* | *Bacillus megaterium* | *Acetobacter aceti* | *Acidovorax avenae* | |
| *Campylobacter coli* | | *lactobacillus* | *Actinobacillus* | *Burkholderia* | *Acetobacter pasteurianus* | *Bacillus megaterium* | |
| *Campylobacter lari* | | *Eubacterium* | *Aeromonas caviae* | | *Agrobacterium larrymoorei* | *Brenneria* | |
| *Campylobacter fetus* | | *prevotella* | *Aeromonas hydrophila* | | *Agrobacterium radiobacter* | *Burkholderia andropogonis* | |
| *Leptospira interrogans* | | *Porphyromonas* | *Aeromonas veronii bovar sobria* | | *Aagrobacterium rhizogens* | *Burkholderia cepacia* | |
| | | *fusobacterium* | *Bordetella bronchiseptica* | | *Agrobacterium rubi* | *Burkholderia gladioli* | |
| | | *Bacillus anthracis* | *Bordetella parapertussis* | | *Agrobacterium tumefaciens* | *Burkholderia glumae* | |
| | | *Bacillus cereus* | *Bordetella pertussis* | | *Agrobacterium ophel* | *Enterobacter cloacae* | |
| | | *Bacillus subtilis* | *Burkholderia cepacia* | | *Bacillus megaterium* | *Enterobacter cloacae subsp dissolvens* | |
| | | *Bacillus thuringiensis* | *Burkholderia pseudomallei* | | *Bacillus pumilus* | *Erwinia amylovora* | |
| | | *Bacillus stearothermophilus* | *Citrobacter freundii* | | *Gluconobacter oxydans* | *Erwinia mallotivora* | |
| | | *Bacteroides fragilis* | *Edwardsiella tarda* | | *Rhizobium rubi* | *Erwinia pyrifoliae* | |
| | | *Brucella suis* | *Enterobacter aerogenes* | | *Sphingomonas melonis* | *Erwinia tracheiphila* | |
| | | *Citrobacter freundii* | *Enterobacter cloacae* | | | *Ewingella americana* | |
| | | *Helicobacter pylori* | *Haemophilus ducreyi* | | | *Janthinobacterium agaricidamnosum* | |
| | | *Neisseria gonorrhoeae* | *Haemophilus haemolyticus* | | | *Pantoea agglomerans* | |
| | | *Neisseria meningitidis* | *Haemophilus influenzae* | | | *Pantoea ananatis* | |
| | | *Staphylococcus aureus* | *Haemophilus parahaemolyticus* | | | *Pantoea stewartii subsp indologenes* | |
| | | *Staphylococcus epidermidis* | *Haemophilus parainfluenzae* | | | *Pantoea stewartii subsp. Stewartii* | |
| | | *Streptococcus equismilis* | *Klebsiella oxytoca* | | | *Pectobacterium atrosepticum* | |
| | | *Streptococcus mutans* | *Klebsiella pneumoniae* | | | *Pectobacterium betavasculorum* | |
| | | *Streptococcus salivarius* | *Kegionella pneumophila* | | | *Pectobacterium carotovorum* | |
| | | *Streptococcus mitis* | *Koraxella catarrhalis* | | | *Pectobacterium wasabiae* | |
| | | | *Morganella morganii* | | | *Pseudomonas amygdali* | |
| | | | *Pasteurella multocida* | | | *Pseudomonas avellanae* | |
| | | | *Plesimonas shigelloides* | | | *Pseudomonas cichorii* | |
| | | | *Proteus mirabilis* | | | *Pseudomonas corrugata* | |
| | | | | | | *Pseudomonas fuscovaginae* | |
| | | | | | | *Pseudomonas mediterranea* | |

**Table S1.** *Cont.*

| | |
|---|---|
| *Proteus vulgaris* | *Pseudomonas meliae* |
| *Providencia alcalifaciens* | *Pseudomonas pseudoalcaligenes* |
| *Providencia rettgeri* | *Pseudomonas savastanoi* |
| *Providencia stuartti* | *Pseudomonas savastanoi* |
| *Salmonella cholerasuis* | *Pseudomonas syringae* |
| *Salmonella enterica* | *Pseudomonas syringae pv. Maculicola* |
| *Salmonella enteritidis* | *Pseudomonas tolaasii* |
| *Salmonella paratyphi* | *Pseudomonas viridiflava* |
| *Salmonella typhi* | *Ralstonia solanacerarum* |
| *Salmonella typhimurium* | *Ralstonia syzygii* |
| *Serratiai marcesans* | *Serratia marcescens* |
| *Shigella boydii* | *Serratia proteamaculans* |
| *Shigella dysenteriae* | *Xanthomonas albilineans* |
| *Shigella flexneri* | *Xanthomonas arboricola* |
| *Shigella sonnei* | *Xanthomonas axonopodis* |
| *Stenotrophomonas maltophilia* | *Xanthomonas campestris* |
| *Vibrio alginolyticus* | |
| *Vibrio cholerae* | |
| *Vibrio fluvialis* | |
| *Vibrio furnisii* | |
| *Vibrio hollisae* | |
| *Vibrio mimicus* | |
| *Vibrio parahaemolyticus* | |
| *Vibrio vulnificus* | |
| *Yersinia enterocolitica* | |
| *Yersinia pestis* | |
| *Yersinia pseudotuberculosis* | |
